# First-in-human intraoperative near-infrared fluorescence imaging of glioblastoma using cetuximab-IRDye800

**DOI:** 10.1007/s11060-018-2854-0

**Published:** 2018-04-06

**Authors:** Sarah E. Miller, Willemieke S. Tummers, Nutte Teraphongphom, Nynke S. van den Berg, Alifia Hasan, Robert D. Ertsey, Seema Nagpal, Lawrence D. Recht, Edward D. Plowey, Hannes Vogel, Griffith R. Harsh, Gerald A. Grant, Gordon H. Li, Eben L. Rosenthal

**Affiliations:** 1 Department of Otolaryngology, Stanford University, Stanford, USA; 2 Department of Radiology, Molecular Imaging Program at Stanford (MIPS), Stanford University, Stanford, USA; 3 Department of Surgery, Leiden University Medical Center, Albinusdreef 2, 2300 RC Leiden, The Netherlands; 4 Department of Neurology, Stanford University, Stanford, USA; 5 Department of Pathology, Stanford University, Stanford, USA; 6 Department of Neurosurgery, Stanford University, Stanford, CA 94305 USA; 7 Stanford Cancer Center, Stanford, CA USA

**Keywords:** Antibody-based imaging, Cetuximab, Brain neoplasms, Fluorescence, Glioblastoma, Image-guided surgery, Phase 1

## Abstract

**Introduction:**

Maximizing extent of surgical resection with the least morbidity remains critical for survival in glioblastoma patients, and we hypothesize that it can be improved by enhancements in intraoperative tumor detection. In a clinical study, we determined if therapeutic antibodies could be repurposed for intraoperative imaging during resection.

**Methods:**

Fluorescently labeled cetuximab-IRDye800 was systemically administered to three patients 2 days prior to surgery. Near-infrared fluorescence imaging of tumor and histologically negative peri-tumoral tissue was performed intraoperatively and ex vivo. Fluorescence was measured as mean fluorescence intensity (MFI), and tumor-to-background ratios (TBRs) were calculated by comparing MFIs of tumor and histologically uninvolved tissue.

**Results:**

The mean TBR was significantly higher in tumor tissue of contrast-enhancing (CE) tumors on preoperative imaging (4.0 ± 0.5) compared to non-CE tumors (1.2 ± 0.3; p = 0.02). The TBR was higher at a 100 mg dose than at 50 mg (4.3 vs. 3.6). The smallest detectable tumor volume in a closed-field setting was 70 mg with 50 mg of dye and 10 mg with 100 mg. On sections of paraffin embedded tissues, fluorescence positively correlated with histological evidence of tumor. Sensitivity and specificity of tumor fluorescence for viable tumor detection was calculated and fluorescence was found to be highly sensitive (73.0% for 50 mg dose, 98.2% for 100 mg dose) and specific (66.3% for 50 mg dose, 69.8% for 100 mg dose) for viable tumor tissue in CE tumors while normal peri-tumoral tissue showed minimal fluorescence.

**Conclusion:**

This first-in-human study demonstrates the feasibility and safety of antibody based imaging for CE glioblastomas.

## Introduction

Glioblastoma has one of the worst prognoses of any human tumor, as evidenced by a median overall survival of 15 months with almost 70% specific annual morbidity [1]. A key variable associated with longer survival is the extent of safe surgical resection [2]. While the infiltrative nature of glioblastoma precludes curative resection, recent studies have provided increasing support for the role of maximal safe resection in extending median survival [2, 3]. With 98% resection and only 1–2 cm^3^ residual volume, the risk of death at any time was shown to be approximately halved compared to patients who did not achieve this extent of resection [3].

The challenge of successfully differentiating normal brain from tumor tissue has put neurosurgery at the forefront of investigating intraoperative navigational systems; however, limitations of current imaging techniques present major obstacles for neurosurgeons in completely removing the diseased tissue because they fail to account for brain shift and for the variability of contrast-enhancement, especially at the tumor margins [4].

To address this issue, recent studies have investigated optical techniques to improve margin detection during resection. Most notably, clinical application using 5-aminolevulinic acid (5-ALA) has shown positive results with 54–70% of cases achieving near-total resection of enhancing tumors [5, 6] and significantly improved progression free survival, but not overall survival [7]. The value of fluorescence-guidance in glioma surgery has recently been acknowledged by the FDA (approved for use in June of 2017). The use of tumor-targeted molecular imaging, rather than metabolic imaging, in the form of near-infrared (NIR) fluorescent dyes labeled to targeted agents has the potential to overcome some of the limitations of 5-ALA [8]. Preclinical data has provided evidence that novel tumor-targeting probes can improve detection, margin control, and survival in a variety of cancer types. In addition, recent clinical studies have demonstrated sensitive and specific cancer detection in fluorescence-guided tumor resections using NIR probes conjugated to therapeutic antibodies in several cancer types [9, 10]. Demonstration that approved antibodies can be repurposed for imaging is important and scalable to other cancer types, since the approval process and cost barriers for fluorescent labeling are limited compared to de novo agents that require costly development and toxicology studies. Recently, cetuximab-IRDye800, targeting the epidermal growth factor receptor (EGFR) in head and neck cancer patients, has been shown to be highly specific for subclinical or non-palpable disease [9]. The clinical value for glioblastoma specifically has been suggested by pre-clinical models [11]. Of note, EGFR is known to be overexpressed in 50–70% of glioblastomas and has shown potential to provide robust tumor delineation from normal tissue due to its relatively low levels of expression in normal brain [12, 13].

We hypothesize that fluorescently labeled antibodies against EGFR will allow for sensitive and specific tumor detection in real-time and can be used safely for the targeted intraoperative detection of primary glioblastoma.

## Methods

### Participants

Patients with suspected glioma were screened for enrollment at Stanford University Medical Center. Inclusion criteria were as follows: (1) subjects undergoing surgical removal as their standard of care, (2) age ≥ 18 years, (3) life expectancy of > 12 weeks, (4) Karnofsky performance status of at least 70%, (5) labs and electrolytes within normal parameters. Exclusion criteria included: (1) received an investigational drug within 30 days prior to enrollment, (2) recent (within 6 months) myocardial infarction, cerebrovascular accident, uncontrolled congestive heart failure or unstable angina, (3) history of infusion reactions to cetuximab or other monoclonal antibody therapies, (4) pregnant or breastfeeding, (5) serious reaction to the loading dose of cetuximab, (6) prolonged QT on ECG or lab values that would preclude resection, and (7) subjects receiving certain antiarrhythmic agents.

### Study design

**Fig. 1:**
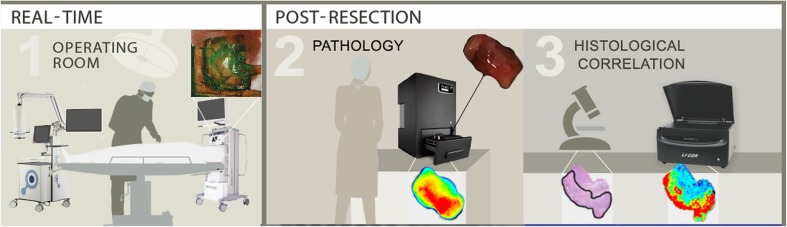
Trial imaging workflow. Real-time fluorescent imaging was performed in the operating room (**1**) using a wide-field NIR imaging device on day 2 following cetuximab-IRDye800 infusion. **2** A closed-field NIR imaging system was used to image the resected specimens ex-vivo. **3** Specimens were sectioned and scanned in surgical pathology using a fluorescence scanning system to correlated fluorescence with histological tissue classification

A dose-escalation model using two doses, 50 or 100 mg of the investigational agent, was chosen to identify the optimal tumor-to-background ratio (TBR). The dosages and timing of infusion were based on the previous study by Rosenthal et al. in which the dose escalations were initially determined by the therapeutic dose of cetuximab (250 mg/m^2^) [9]. In the same study, TBRs were measured at varying time points starting on the day of infusion and while the half-life of the study drug is known to be approximately 24 h, the TBR was highest on the day of surgery around day 3. This informed our decision to infuse the study drug 2–5 days prior to surgery. Informed consent was obtained from all individual participants included in the study. A 100 mg pretreatment dose of unlabeled cetuximab was administered to patients before the study drug both to differentiate potential reactions between a cetuximab reaction and a cetuximab-IRDye800 reaction, and to saturate normal tissues with a high expression of EGFR (antigen sinks, e.g. liver) [14]. Baseline hemodynamic measurements and ECG data were used to compare changes in these parameters after loading dose infusion. Following a 3-h observation period monitoring for adverse reactions after administration of the loading dose, the study drug was infused. Patients were then observed for an additional 3 h, hemodynamic measurements and ECGs were repeated, then patients were discharged home. 2 days after cetuximab-IRDye800 infusion, the three enrolled patients underwent surgical resection. During intraoperative imaging, the protocol stipulated that the imaging data would not be used to influence the surgical procedure. The Stanford University Institutional Review Board and the FDA approved the study protocol and the study was registered in ClinicalTrials.gov, under number NCT02855086. All procedures performed were in accordance with the ethical standards of the institutional and/or national research committee and with the 1964 Helsinki declaration and its later amendments or comparable ethical standards. Adverse events were classified according to the National Cancer Institute Common Terminology Criteria (Version 4.0) and were recorded for up to 30 days beyond the infusion of cetuximab-IRDye800. The overall workflow of the study is outlined in Fig. 1.

### Investigational agent: cetuximab-IRDye800

The cetuximab-IRDye800 was manufactured under cGMP conditions at the UAB Vector Production Facility as previously described [15].

### Optical NIR imaging during surgery

Imaging during surgery was performed using the wide-field optical imaging system PINPOINT (Novadaq, Burnaby, Canada), and the wide-field SurgVision Explorer Air (SurgVision BV, ‘t Harde, The Netherlands). Both wide-field systems were used for NIR fluorescence imaging of the tumor first following tumor exposure and again after resection to assess the wound bed for any residual fluorescence. Fluorescence images were displayed in real time and a pseudo-colored (lime green) merged image of the color video and fluorescence image was generated. Subsequently, all excised tissues were imaged ex vivo on the OR back table immediately following removal using both wide-field systems. Next, the surgical specimen was processed by the pathologist according to standard clinical practice.

### Ex vivo closed-field NIR imaging

The Pearl Impulse imaging platform (LI-COR Biosciences, Lincoln, NE) was used to image fresh tissues obtained in the operating room. The system was used to measure NIR-fluorescent signals of the tumor as mean fluorescence intensity (MFI), defined as total counts/region of interest (ROI) per pixel area, and to calculate TBRs using the system’s integrated instrument software (ImageStudio, LI-COR Biosciences). The background signal was defined by peri-tumoral tissue samples removed for surgical exposure of the tumor and later confirmed to be histologically normal by a neuropathologist. After measuring the signal intensities as MFIs, TBRs were calculated for each patient’s primary tumor specimen using the following formula:

$$TBR{\text{ =}}\frac{{MFI{\text{ }}of{\text{ }}primary{\text{ }}tumor}}{{MFI{\text{ }}of{\text{ }}histologically{\text{ }}negative{\text{ }}tissue}}.$$

### Pathologic assessment

Formalin-fixed paraffin-embedded tumor tissues were sectioned at 5 µm thickness and fluorescence imaging was performed using the Odyssey NIR scanner (Li-COR Biosciences). In addition to being scanned using the Odyssey NIR scanner, all histologic sections were stained with standard hematoxylin–eosin immunohistochemical staining (H&E) so that representative tissue sections from each specimen could be assessed by a neuropathologist for tumor status—the current gold standard for tumor determination. To confirm the presence of EGFR, additional sections were stained with immunohistochemistry (IHC) for anti-human EGFR (predilute, rabbit, clone 5B7, 790-4347, Ventana, Tucson, AZ). Automated immunohistochemical staining was performed with Ventana Benchmark XT (Ventana Medical Systems, Inc., Tucson, AZ, USA).

### Statistical analysis

Graphpad Prism (version 23.0, IBM Corp.) was used for statistical analyses. Differences in fluorescent signal per tissue type (tumor and histologically normal or necrotic tissues) were tested against one another and also between different doses, by an independent sample Student *t* test. Sensitivity, specificity, and negative and positive predictive values (NPV and PPV) were determined by assigning MFIs to individual boxes on a grid overlying the fluorescently scanned slides and also assigning each box as “viable tumor” or “non-viable tumor/normal tissue” as determined by an experienced neuropathologist who was blinded to fluorescence status. The average MFI of all boxes that did not include viable tumor tissue was calculated and set as a threshold for fluorescence positivity. As this is the first in-human study using this technology, it was important to establish a baseline of non-specific fluorescence uptake in non-tumor tissue. This methodology has previously been demonstrated in prior publications in a variety of tumor types [16, 17]. A histologically proven tumor specimen that was fluorescent was considered a true-positive; a histologically normal specimen that was fluorescent was a false-positive; a histologically normal specimen that was non-fluorescent was a true-negative; and a histologically positive tumor that was non-fluorescent was a false-negative. Sensitivity and specificity were subsequently calculated using these definitions and reported as 95% confidence intervals.

## Results

### Participants

Between July 2016 and April 2017, three patients received the study drug. Four patients were screened for eligibility, and three were enrolled. One patient had signed consent suffered a seizure and subsequent somnolence prior to enrollment in the clinical trial and therefore did not meet inclusion criteria. Two patients received 50 mg cetuximab-IRDye800 (low dose) and one patient received 100 mg (high dose). Tumor size as determined by pre-operative imaging ranged from 1.5 to 8.0 cm in diameter. On MRI, two patients had contrast-enhancing (CE) tumors, one of whom received 50 mg cetuximab-IRDye800 and the other received 100 mg, and one patient had a non-enhancing tumor and received 50 mg cetuximab-IRDye800. The two enhancing tumors were later determined by pathology to be glioblastomas while the non-enhancing tumor was determined to be a Grade II diffuse astrocytoma.

### Safety data

There were no related grade-2 adverse events to cetuximab-IRDye800, and one possibly related grade-1 adverse event as one patient had an elevated alanine aminotransferase (ALT) following infusion. The QTc interval increased after infusion of the unlabeled cetuximab loading dose for two of three patients, as expected, and returned to baseline after infusion of cetuximab-IRDye800.

### Intra-operative NIR fluorescence imaging

**Fig. 2:**
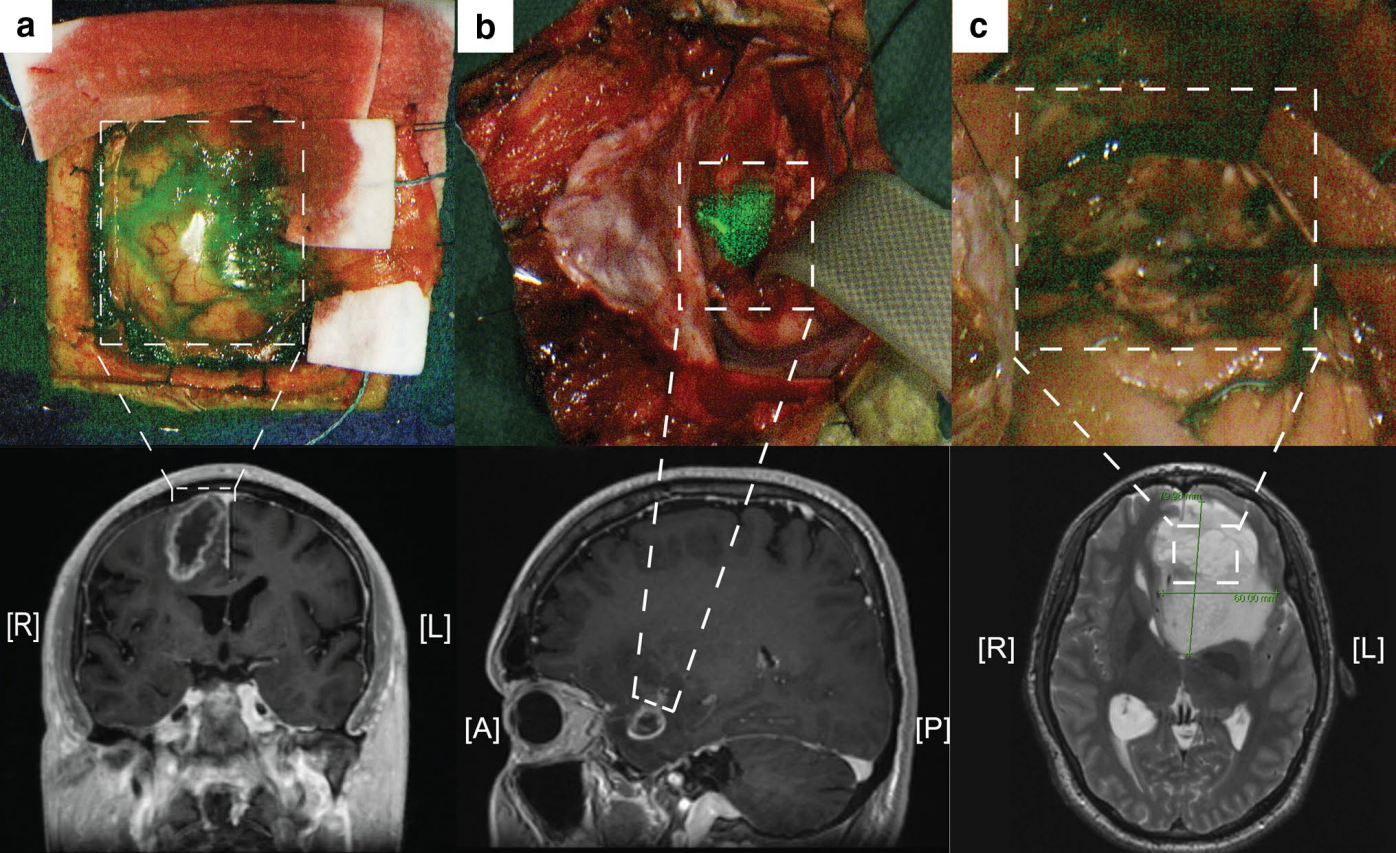
Representative intraoperative fluorescent images and associated pre-operative Magnetic Resonance Images (MRIs). Fluorescence image following tumor exposure in **a** patient 1, **b** patient 2, and **c** patient 3 and associated MRIs below

Intraoperative NIR fluorescence was detected in two of the three enrolled patients. The two patients with CE tumors on pre-operative MRI showed intraoperative fluorescence (Fig. 2a, b). In the one non-enhancing tumor no fluorescence signal was detected intraoperatively (Fig. 2c) and therefore this patient was excluded from statistical analyses.

### Threshold of detection

**Fig. 3:**
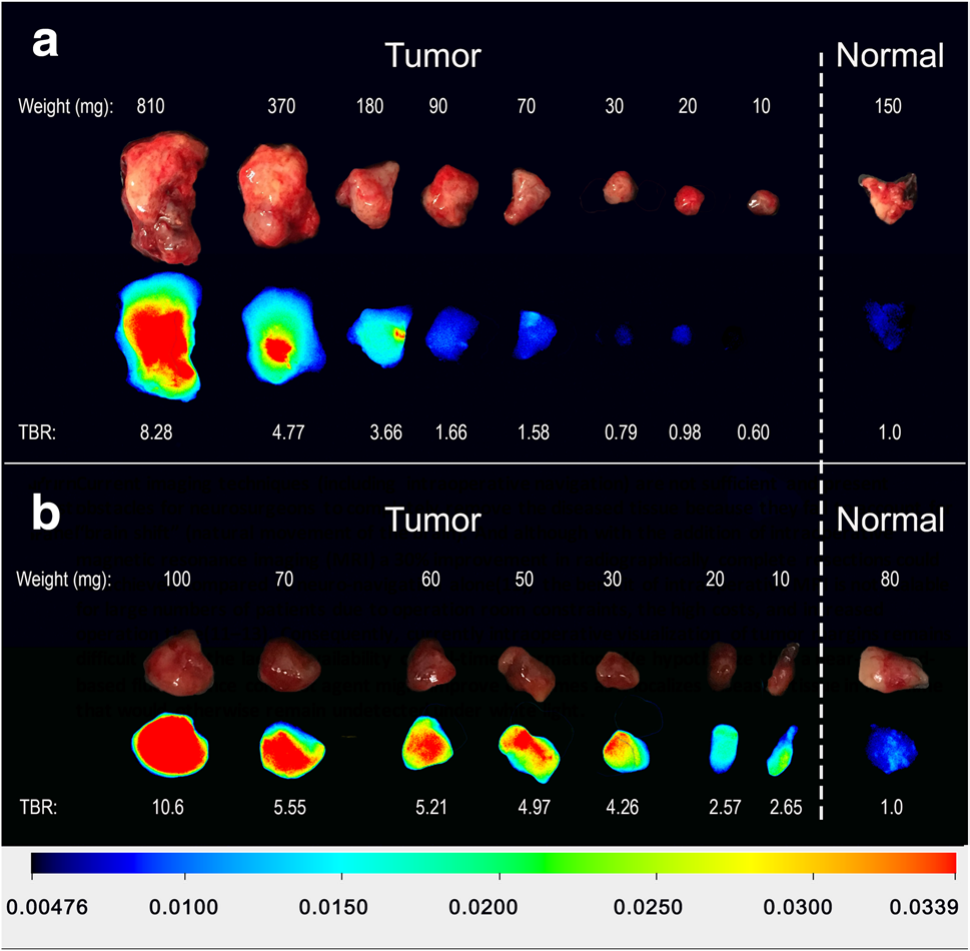
Fluorescent images of serially cut fresh tumor and histologically normal tissue imaged in the closed-field setting. Serial sections from **a** patient 1 and **b** patient 2. Minimal detectable tumor weights were found to be 70 and 10 mg for patient 1 (low dose) and patient 2 (high dose), respectively

The smallest histologically-confirmed tumor fragment that could be detected by NIR fluorescence imaging was determined for each dose level and was measured by TBR in a closed-field setting. A TBR greater than one meant that the signal in tumor tissue was greater than that of normal tissue and was therefore classified as “detectable.” In the low dose CE patient, the lowest detectable tumor weight was 70 mg (TBR of 1.58) (Fig. 3a) versus 10 mg (TBR 2.65) in the high dose patient (Fig. 3b). In the non-CE patient the tumor tissue fluorescence was not detectable compared to normal tissue (images not shown).

### Correlation of NIR fluorescence ex vivo with histological disease

**Fig. 4:**
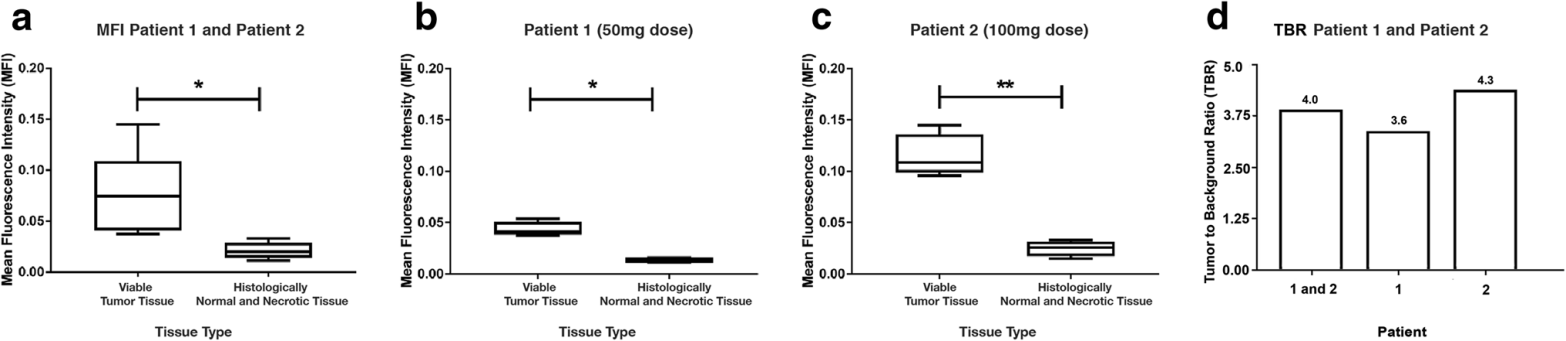
MFI of tumor tissue compared to histologically normal and necrotic tumor tissue in glioblastoma patients. **a** MFI for patient 1 and patient 2 combined, **b** MFI of patient 1 only (low dose patient), **c** MFI of patient 2 only (high dose patient) (*P < 0.01, **P < 0.001), **d** TBR of patient 1 and 2 combined, patient 1 alone, patient 2 alone

To evaluate the correlation between the NIR fluorescence signal and histologic evidence of disease, NIR fluorescence imaging and pathologic assessment of the primary specimen and histologically normal tissue were compared. Fluorescence was highly specific for viable tumor tissue in patients with enhancing tumors; histologically normal peri-tumoral tissue showed minimal fluorescence. The MFI for tumor tissue was 0.08 ± 0.014 compared to 0.02 ± 0.003 (p < 0.01) for normal brain or necrotic tumor tissue in both patients combined (Fig. 4a), 0.04 ± 0.004 versus 0.01 ± 0.002 in the low dose patient (p < 0.01) (Fig. 4b), and 0.11 ± 0.011 versus 0.02 ± 0.004 in the high dose (p < 0.001) (Fig. 4c). The mean TBR for the two CE tumors combined was 4.0 ± 0.5, and when separated the higher dose (100 mg) led to a higher TBR compared to the lower dose (50 mg) (4.3 vs. 3.6) (Fig. 4d).

### Histological correlation

**Fig. 5:**
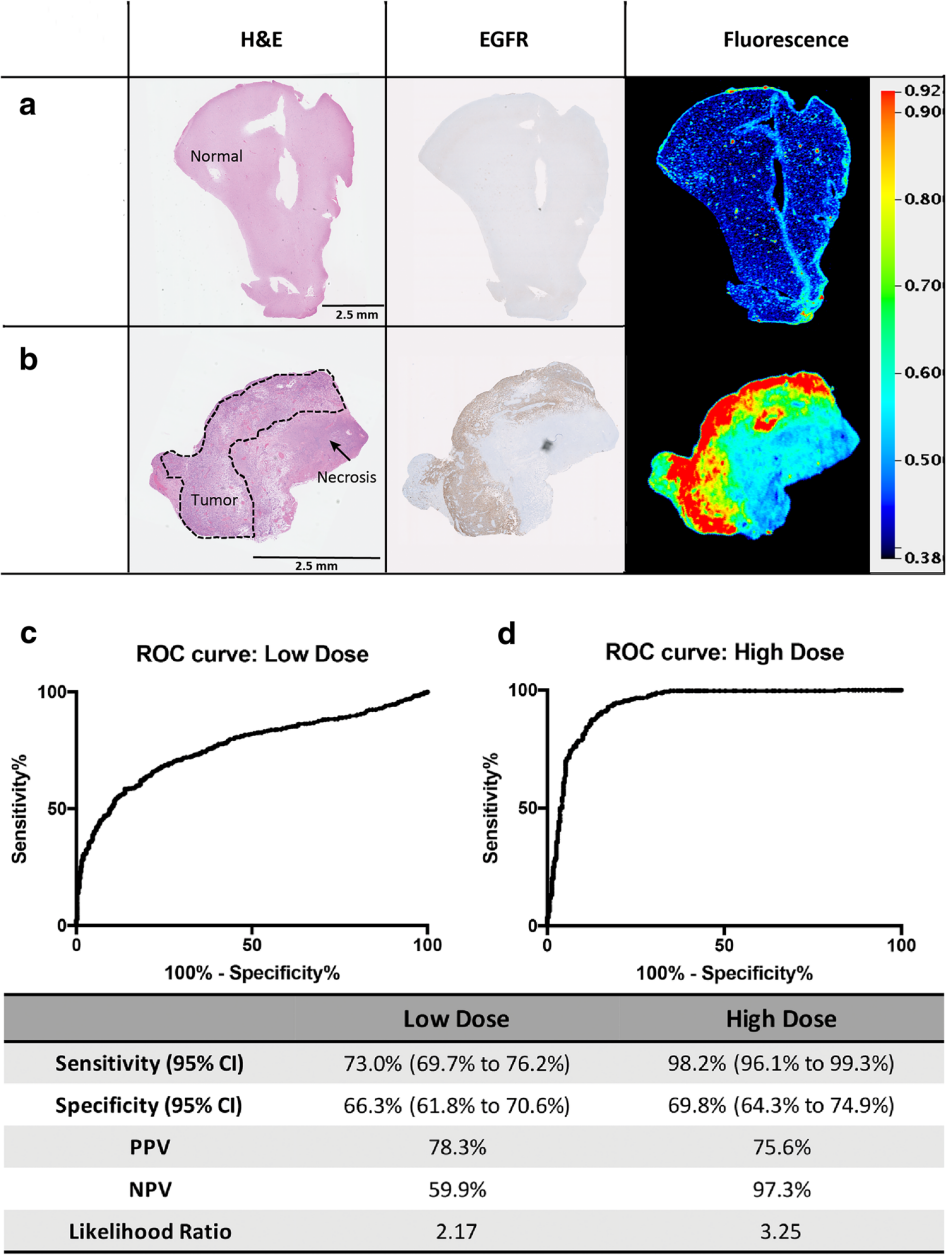
Representative images and statistical analysis of histological correlation with fluorescence. H&E stain, EGFR and fluorescent images of representative sections containing histologically normal tissue (**a**) and sections containing tumor and necrotic tissue (**b**). ROC curves for low (**c**) and high (**d**) dose CE glioblastoma patients and associated sensitivity and specificity calculations

EGFR expression was decreased in normal brain tissue (Fig. 5a) and also in necrotic tumor tissue (Fig. 5b) compared to viable tumor tissue (Fig. 5b). To assess tumor specificity, histological co-localization of the antibody-dye was performed to correlate antibody deposition in the tumor and determine off-target uptake of the probe using the methods described above in which all analyzed tissue sections were included. Viable tumor tissue could be identified in the low dose patient with a sensitivity of 73.0% (CI 69.7–76.2), a specificity of 66.3% (CI 61.8–70.6), AUC of 0.76 (CI 0.74–0.79), and a PPV of 78.3 and 59.9% NPV (Fig. 5c). In the high dose patient, a sensitivity of 98.2% (CI 96.1–99.3) was achieved, with a specificity of 69.8% (CI 64.3–74.9), AUC of 0.94 (CI 0.92–0.96), and PPV and NPV of 75.6 and 97.3%, respectively (Fig. 5D).

## Discussion

Tumor-specific molecular imaging using cetuximab-IRDye800 was demonstrated to be both safe and feasible for intraoperative visualization of glioblastoma and provided a clear differentiation between tumor and surrounding histologically normal brain tissue in patients with contrast enhancing tumors on preoperative MRI. Tumor tissue samples weighing as little as 10 mg were distinguishable from histologically normal brain tissue samples during ex vivo imaging, warranting further investigation of the dye’s ability to differentiate similarly small amounts of tumor tissue intraoperatively. Despite positive expression of EGFR by immunohistochemistry, the non-CE tumor did not demonstrate NIR fluorescence after cetuximab-IRDye800 administration. This is similar to what has been reported using other intraoperative contrast agents; patients are excluded from 5-ALA fluorescence-guided surgery if preoperative contrast-enhanced MRI shows no accumulation [7]. In both cases, the blood–brain barrier is likely to play a critical role. Moreover, it is known that high-grade gliomas generally demonstrate more contrast-enhancement than do low-grade gliomas [18]. Therefore, it is not surprising that the study patient with a non-enhancing tumor (grade II diffuse astrocytoma) did not demonstrate an accumulation of cetuximab-IRDye800, suggesting that decreased permeability may prevent the agent from reaching the tumor regardless of EGFR expression. In contrast, in both patients with CE gliomas on preoperative MRI, intraoperative NIR fluorescence was seen. Whether or not cetuximab-IRDye800 can be of value for intraoperative identification of low-grade gliomas remains to be determined.

Current imaging techniques present obstacles for neurosurgeons to completely remove the diseased tissue, in part because they fail to account for anatomical changes during resection. While the addition of intraoperative MRI resulted in a 30% improvement in radiographically complete resections compared to neuro-navigation alone, the benefit of intraoperative MRI is not scalable for large numbers of patients due to operating room constraints, high costs, and increased operation time [19–21]. Consequently, surgeons do not have access to good real-time information during oncologic surgery. Although 5-ALA is approved and can be used in real time it relies on a longer wavelength and is associated with limitations discussed below. These limitations underscore the potential utility of a near infrared fluorescence contrast agent that lacks many of the aforementioned constraints. The infrastructure required to utilize cetuximab-IRDye800 is largely already in place in many major hospitals, as the excitation and emission wavelengths overlap with indocyanine green (ICG), thus eliminating the need to purchase or train surgeons on new imaging devices during initial implementation of the technology.

Successful delineation of tumor from normal tissue remains the critical measure of a successful contrast agent. Although 5-ALA is currently the only clinical agent available for real-time imaging during brain tumor resection, there are several key properties that are likely to limit the success of 5-ALA compared to a NIR contrast agent. Most notably, 5-ALA demonstrates limited tissue penetration in part due to blood being a strong absorbing material for both the excitation and emission wavelengths for 5-ALA fluorescence. Similarly, other dyes including indigo carmine, ink and ICG have also been suggested to affect the detection of 5-ALA which prevents its use in augmenting other well-established techniques in assisting with tumor resection [22]. The wavelengths inherent to NIR contrast agents avoid these limitations. Non-malignant radiation necrosis or neurodegenerative disease have also been previously reported to appear fluorescent using 5-ALA, suggesting that inflammation and reactive cell turnover may be a source of false positive fluorescence resulting in removal of non-tumor tissue [23]. Another disadvantage of imaging with 5-ALA is dependence on high tumor metabolic rates that result in intratumoral and intertumoral heterogeneity of PpIX expression [24]. These studies have reported up to 5 log-orders of difference in PpIX levels between patients, and currently no imaging system is available to image such a wide range of intensities. We have demonstrated that significant TBRs can be achieved even with variation in expression of EGFR. Moreover, we have demonstrated that a higher dose of cetuximab-IRDye800 leads to improved delineation between tumor and normal tissue, which is consistent with the aforementioned study in squamous cell carcinoma (SCC) with this agent [9], where increased doses led to higher signals in the primary tumor. However, in the case of SCC, the dose limiting factor was based on false-positive results in lymph nodes at higher doses [16]. The increased sensitivity and specificity seen in the high dose as compared to the low dose patient in the current study suggests that false-positivity is less of a concern in glioblastoma resection, though our small sample size warrants further study. It is possible that even higher doses would improve TBRs.

## Limitations

While this study represents a successful first in human proof-of-concept of an antibody-based strategy for intraoperative imaging during glioma resection, important limitations to the current study should be addressed. The small sample size represents the most important limitation in drawing conclusions regarding this technique’s clinical utility. While high sensitivities and specificities were obtained, each dose was analyzed in only one patient each, preventing an evaluation of its consistency across patients. Thresholds for negative fluorescence in each dose were based on only one patient in each dose, which limits the conclusions that can be drawn regarding the sensitivity and specificity to only the two patients included in the trial. In the larger study currently in preparation, these thresholds will be set by an average of all patients who receive a given dose of study drug. In addition, the EGFR expression status for each patient was unknown prior to undergoing resection. While the internalization and fast EGFR turnover in tumor tissue leads to increased fluorescent signal due to dye accumulation inside tumor cells, variability in expression status may affect fluorescence accumulation and therefore fluorescent signal. It is also challenging to directly relate the diseased tissue shown on preoperative MRI to the resected tissue in order to show direct correlation with the current standard of care using preoperative imaging. Despite these limitations, this study demonstrates an important proof-of-principle of a new technique, warranting further investigation in more patients in future studies currently in preparation.

## Conclusion

While fluorescence imaging during surgery is in its early stages of development, we have demonstrated that it can be safely used for contrast enhancing high-grade glioblastomas and that the antibody-dye conjugate is specific to brain tumor tissue. The implementation of this technology has been shown to be feasible for neurosurgical resection of brain tumors with minimal disruption to the workflow and standard of care. Antibody based imaging has the potential to maximize the extent of primary tumor resection and identify residual disease after resection.
